# Integrated Analysis of Transcriptome in Cancer Patient-Derived Xenografts

**DOI:** 10.1371/journal.pone.0124780

**Published:** 2015-05-07

**Authors:** Hong Li, Yinjie Zhu, Xiaoyan Tang, Junyi Li, Yuanyuan Li, Zhaomin Zhong, Guohui Ding, Yixue Li

**Affiliations:** 1 Key Laboratory of Systems Biology, Institute of Biochemistry and Cell Biology, SIBS, CAS, 320 Yueyang Road, Shanghai, 200031, China; 2 Shanghai High School, Shanghai, 200231, China; 3 Shanghai Center for Bioinformation Technology, 1278 Keyuan Road, Shanghai, 201203, China; Taipei Medicine University, TAIWAN

## Abstract

Patient-derived xenograft (PDX) tumor model is a powerful technology in evaluating anti-cancer drugs and facilitating personalized medicines. Multiple research centers and commercial companies have put huge efforts into building PDX mouse models. However, PDX models have not been widely available and their molecular features have not been systematically characterized. In this study, we provided a comprehensive survey of PDX transcriptome by integrating analysis of 58 patients involving 8 different tumors. The median correlation coefficient between patients and xenografts is 0.94, which is higher than that between patients and cell line panel or between patients with the same tumor. Major differential gene expressions in PDX occur in the engraftment of human tumor tissue into mice, while gene expressions are relatively stable over passages. 48 genes are frequently differentially expressed in PDX mice of multiple cancers. They are enriched in extracellular matrix and immune response, and some are reported as targets for anticancer drugs. A simulation study showed that expression change between PDX and patient tumor (6%) would result in acceptable change in drug sensitivity (3%). Our findings demonstrate that PDX mice represent the gene-expression and drug-response features of primary tumors effectively, and it is recommended to monitoring the overall expression profiles and drug target genes in clinical application.

## Introduction

Patient-derived xenograft (PDX) is developed by surgically implanting tumor tissue directly from a patient biopsy into an immunodeficient mouse. When Successful xenograft tumors (F1 generation) grow to 1 cm^3^, they are removed and passaged to more immunodeficient mice. This progress expands the original human tumor to multiple mouse passages, providing an enriched resource for studying tumor biology and evaluating anticancer drugs. Previous studies have successfully built PDX models for breast cancer, lung cancer, liver cancer, colon cancer and so on. The average time for generating progressively growing xenograft tumor model ranges from 2 to 12 months [1]. The engraftment rate ranges from 23% to 75% depending on tumor types [1]. Advanced tumors and patients with poor survival intend to have higher engraftment rate [2].

Comparison of gene expression profiles between human pancreatic ductal adenocarcinoma and their PDXs suggested that mouse xegnografts were highly similar with the original tumors in gene expression pattern [3]. A study of non-small cell lung cancer (NSCLC) revealed that chemotherapeutic responsiveness of the PDXs resembled the clinical situation in NSCLC [4]. Ding et.al compared a primary breast cancer, its brain metastasis, and primary-derived PDX by whole genome sequencing; their results demonstrated that PDX retained all primary tumor mutations and displayed mutations in the metastatic sample [5]. Further sequencing of 17 originating tumors, xenograft, and germ-line trio DNA confirmed that the genome-wide variant allele frequency (VAF) was often preserved in PDX models, and the correlation coefficients of VAF varied from 0.32 to 0.86 [6]. These studies demonstrated that PDX models recapitulate the genetic, molecular, and morphological characteristics of human tumors [3–8]. Additionally, PDXs partly reproduce the tumor microenvironment and tumor cell interactions, which are lost in tumor-derived cell lines [1,9]. Therefore, PDX model is thought to be more promising than cancer cell lines for studying cancer mechanism and drug effects [10] [11]. Oncotest et al. tested 11 cytotoxic anticancer agents in ~200 tumor xenografts and revealed a set of gene signatures for predicting tumor response [12]. In a clinical trial of 14 patients with advanced cancers, mouse xenografts were treated with 63 different drugs and the effective treatments were identified for 12 patients [13].

However, there still exist differences between the PDX and the original tumor. For example, PDX-specific somatic mutations were observed in breast-cancer-derived xenografts, which may be passenger mutations randomly selected during transplantation or mutations selected by increasing tumor fitness [6]. Regarding the microenvironment, human stromal cells are substituted by the mouse stromal cells in PDX, which might affect tumor growth [14]. Therefore, it is necessary to identify the molecular difference between PDX and original tumor and explore how the difference affects drug response.

Here we investigated the genome-wide expression similarity between cancer patients and PDXs by meta-analysis of multiple cancer types, and explored the potential effect of expression change on drug response. The median correlation coefficient between patients and xenografts is 0.94, better than cancer cell lines to recapitulate the expression pattern of original tumor. We found that the main expression change occurred in the engraftment of human tumor tissue into mice, and gene expressions were more stable in mice over passages. Meta-analysis of multiple cancer types revealed that some genes, which were frequently differentially expressed in the mouse xenografts, were highly enriched in extracellular matrix and immune response. Drug sensitivity got from PDX generally recapitulates the real drug sensitivity in patient tumor, except in specific situations, such as when gene expressions of PDX mouse are hugely changed or drug target genes are significantly differential.

## Methods

### Gene expression datasets

We searched GEO database by keywords “tumor” and “xenograft”, only keeping datasets with expression data from both patient biopsy and mouse xenograft. Totally we collected 9 datasets involving 8 tumor types. Expression matrix and microarray platform information were downloaded by R package “GEOquery”. Expression matrix was quartile normalized and log-transformed for preprocessing [15]. Gene expression values were set as the median of probes’ expression levels.

### Comparison of cancer patient biopsy and PDX

We manually matched the patient-xenograft pairs by checking sample descriptions and related publications. Samples from cancer patient biopsy were labeled as F0, mouse xenografts were labeled as F1, F2 …… (Unknown passages were labeled by F?). Expression similarity was evaluated by Spearman's rank correlation coefficient (SRCC). Fold change was calculated for each “human tumor VS. xenograft” and “xenograft VS. xenograft” pair, and cutoff 1.5 was used to screen differentially expressed genes.

Functional enrichment in GO and KEGG databases were performed by DAVID [16]. Interacting drugs were searched from database ‘cancerresource’ (http://bioinf-data.charite.de/cancerresource). Drug targets were got from DrugBank V4.1 [17].

### Comparison of different cancer models

We compared three methods for evaluating anticancer drugs: 1) patient-derived xenograft, 2) cancer cell line, 3) tumor tissues form other patients. We only considered patients who had PDX model and their corresponding cancer types had available samples in 2) and 3) (S3 Table). Expression of cancer cell lines were obtained from GDSC (Genomics of Drug Sensitivity in Cancer), a database contains gene expression and drug sensitivity data (half maximal inhibitory concentration, IC50) for 138 anticancer drugs across almost 700 cancer cell lines [18]. Expression data of human tumor tissues were downloaded from GSE2109 (http://www.ncbi.nlm.nih.gov/geo/query/acc.cgi?acc=GSE2109), which consisted of 2158 arrays from solid tumors. When combining datasets from different studies, it is necessary to standardize data to remove batch effect [19,20]. Therefore, we kept genes available on all arrays, and performed the between-study normalization by “ComBat” function in R.

### Prediction of drug response from expression data

A widely used anticancer drug, cisplatin, was selected as an example to evaluate drug response. Gene expression and cisplatin IC50 data for 497 cancer cell lines were downloaded from GDSC database. To simulate expression change in PDX mice, we randomly generated simulation datasets from the GDSC expression data. Fold change of gene expression profiles were simulated from a normal distribution (ND), and then multiplied by GDSC data to get simulated expression data. We generated 10500 simulation datasets by changing the standard deviation (SD) of ND from 0 to 0.2 and randomly generating ND 500 times for each SD. SRCC between simulated and real expression profiles was calculated for each cell line, and their median was used to measure the expression change in simulation data.

Paul et al. developed a ride regression method which predicts clinical drug response from gene expression level [21]. We used this method to train a model from real GDSC expression and cisplatin IC50 data [18]. The SRCC between real IC50 and predicted IC50 were called “SRCC_IC50”. We applied the prediction model to simulated expression datasets and calculated “SRCC_IC50”. To remove the effect of prediction model and evaluate the difference in drug sensitivity effectively, “SRCC_IC50” was divided by 0.84, which is the value of “SRCC_IC50” when we applied the prediction model to training data.

## Results

### Expression similarity between cancer patients and PDXs

We downloaded and normalized 9 PDX expression datasets from GEO databases, including hepatocellular carcinoma, colorectal cancer, breast cancer, pancreatic ductal adenocarcinoma, head and neck squamous cell carcinoma, adenoid cystic carcinoma, acute lymphoblastic leukaemia, and lung cancer (Table 1). Meta-analysis of these 9 datasets was performed to systematically understand the expression similarity between cancer patients and PDXs. Totally, 58 patients have expression data from PDX mice, the PDX passage varied from 1 to 16. Spearman's rank correlation coefficient (SRCC) was used to compare the 56 “human tumor VS. xenograft” pairs and 23 “xenograft VS. xenograft” pairs.

**Table 1:** GEO expression datasets used in this study.

GEO expression dataset	Platform	Cancer Type	Number of cancer patient	Number of “human tumor VS. xenograft” pair	Number of “xenograft VS. xenograft” pair	Max passages of PDX
GSE6465	GPL570	Hepatocellular Carcinoma	7	7	0	?
GSE55828	GPL15207	Hepatocellular carcinoma	9	9	0	3
GSE35144	GPL570	colorectal cancer	14	19	5	14
GSE46106	GPL570	breast cancer	1	0	4	15
GSE45153	GPL570	Head and neck squamous cell carcinoma	5	4	3	10
GSE28996	GPL570	Adenoid cystic carcinoma	5	5	0	16
GSE46385	GPL570	pancreatic ductal adenocarcinoma	5	1	10	12
GSE57491	GPL6884	paediatric B-cell precursor acute lymphoblastic leukaemia	9	9	0	?
GSE15240	GPL570	Small Cell Lung Cancer	3	2	1	2

**Fig 1:**
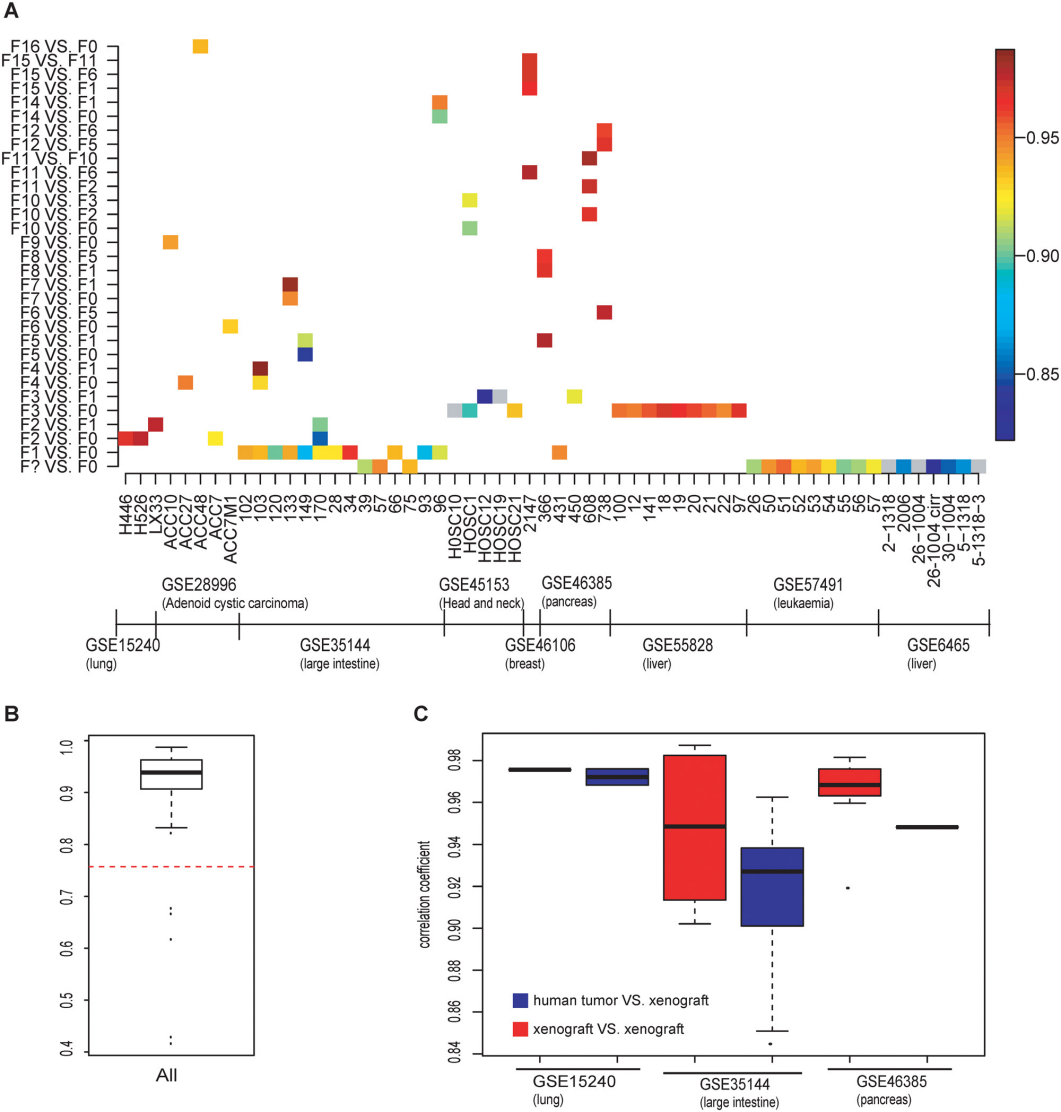
Expression correlation analysis for human cancer patients and PDX mouse models. (A) Heatmap showing the Spearman's rank correlation coefficient (SRCC) of 8 cancers in 9 GEO datasets. F0 indicates cancer patient biopsy, F1 is the 1^st^ passage PDX, F2 is the 2^nd^ passage PDX, …, F? is the PDX whose passage is unclear. (B) Boxplot showing the distribution of SRCC. The red line is the mean minus 1.5 standard deviations. (C) Comparison of the similarity between “human tumor VS. xenograft” (blue) and “xenograft VS. xenograft” (red).

Fig 1A illustrates the heatmap of SRCC in multiple kinds of comparisons and different cancer datasets. The median SRCC of different cancer datasets varies from 0.82 to 0.97. Correlations between most tumor patients and PDX are higher than 0.9, even for >10-generation PDXs. The distribution of all SRCC is shown in Fig 1B. Its mean is 0.91, median is 0.94, and standard deviation is 0.10. SRCC below 0.76 (mean minus 1.5 standard deviation) is regarded as the outlier. There are 5 outliers among 79 comparisons and they only exist in GSE45153 and GSE6465. We ignored these 5 outliers in the subsequent analyses. Then we focused on three datasets (GSE15240, GSE35144, GSE46385) with both “human tumor VS. xenograft” and “xenograft VS. xenograft” pairs (Fig 1C). The SRCCs in “human tumor VS. xenograft” pairs are significantly lower than SRCCs in “xenograft VS. xenograft” pairs (Wilcox test P = 0.0005). It suggests that expression change between the human cancer tissue and PDX model is much larger than the change between PDXs. Differential gene expressions in PDX are majorly in the engraftment of human tumor tissue into mice while the gene expressions of PDX are robust through multiple transplantations.

### Common differentially expressed genes shared among different cancer types

**Fig 2:**
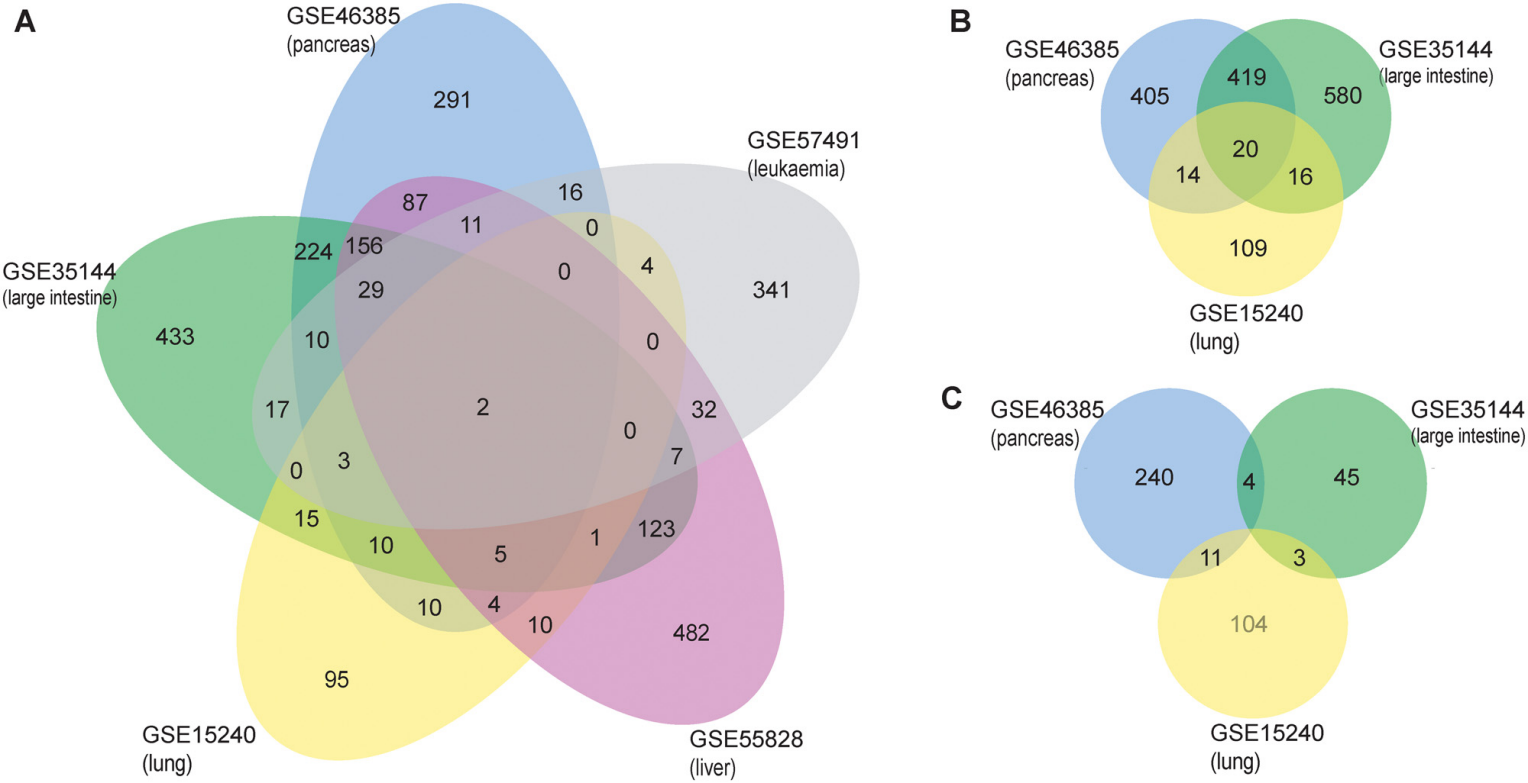
Differentially expressed genes in multiple cancer datasets. (A). Genes in “human tumor VS. xenograft” comparisons in five datasets. (B) Genes in “human tumor VS. xenograft” comparisons, only using three datasets in the same platform GPL570. (C) Genes in “xenograft VS. xenograft” comparisons.

We screened differentially expressed genes in “human tumor VS. xenograft” comparisons, using five expression datasets from three microarray platforms: GPL570 (GSE46385, GSE35144, GSE15240), GPL6884 (GSE57491) and GPL15207 (GSE55828). The number of up-regulated genes ranges from 2 to 76, number of down-regulated genes ranges from 14 to 656 (S1 Table). Fig 2A shows the number of genes that are differentially expressed in at least one paired comparison in each dataset. Some differentially expressed genes are shared in multiple datasets, especially for the three datasets from same GPL570 platform (Fig 2B). We also compared differentially expressed genes in “xenograft VS. xenograft” pairs. The number of up-regulated genes ranges from 1 to 55, number of down-regulated genes ranges from 1 to 91 (S1 Table). Very few genes are simultaneously changed in the “xenograft VS. xenograft” pairs in two datasets (Fig 2C).

**Fig 3:**
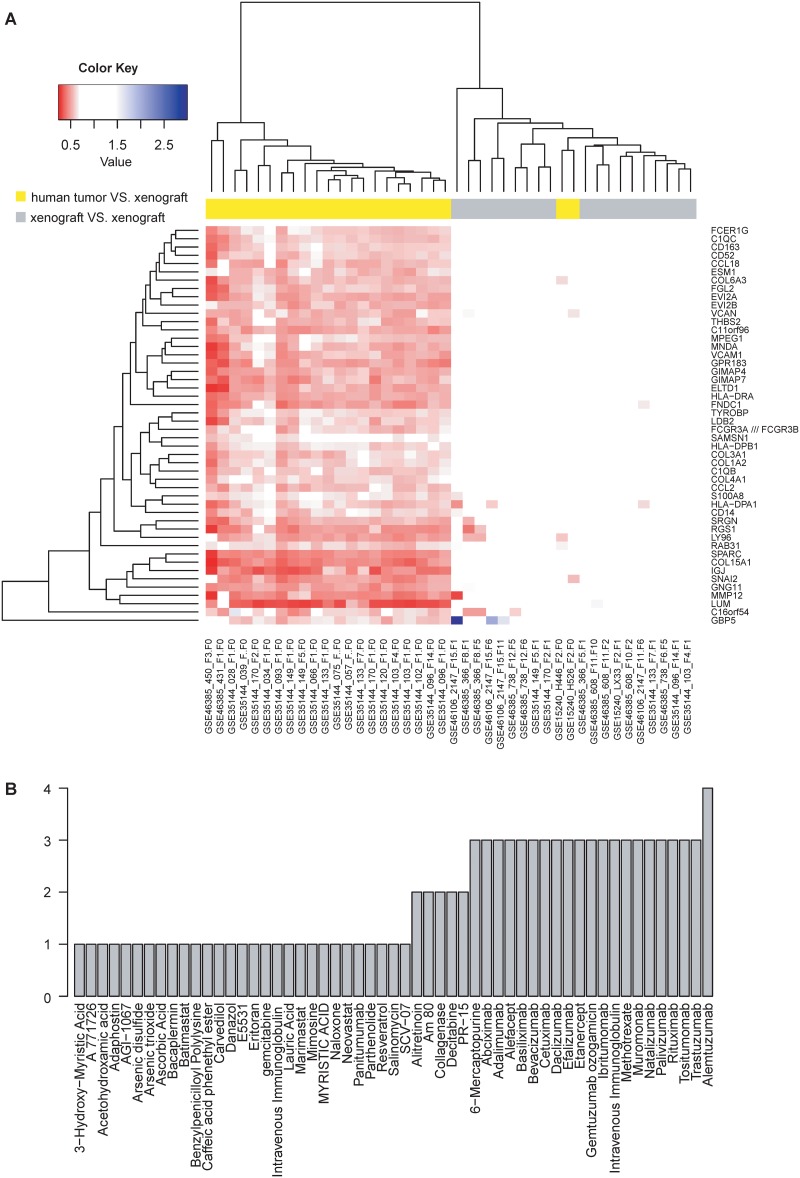
Common differentially expressed genes in PDX mice and their interacting drugs. (A) Expression heatmap of 48 genes, which were differentially expressed in more than half cancer patients and more than two cancer types. (B) Number of differentially expressed genes that have interaction with anticancer drugs.

To understand the common change of PDXs in different cancers and patients, we selected common differentially expressed genes between human tumor biopsy and PDXs. There are 48 genes that are differentially expressed in more than two GSE datasets and more than half cancer patients (S2 Table). Expression heatmap of these genes is shown in Fig 3A. Almost all “human tumor VS. xenograft” pairs are clustered together (except GSE15240) and all “xenograft VS. xenograft” pairs are in another cluster. Since GSE15240 have only two “human tumor VS. xenograft” pairs and the number of differentially expressed genes for these two pairs is smaller than the number of most “human tumor VS. xenograft” pairs in other datasets, GSE15240 is not clustered with others.

To evaluate whether common differentially expressed genes shared among different cancer types may be caused by some dominant data, we removed one dataset and selected genes from the remained four datasets each trial. We obtain 45, 25, 57, 106, and 73 genes when removing GSE46385, GSE35144, GSE15240, GSE57491 or GSE55828, respectively; 15 genes remain in all conditions (S1A Fig). Among 48 common differentially expressed genes, 15 genes are kept no matter which dataset is removed, and others are kept in more than 3 conditions (S2 Table). GSE35144 is the dominant data, whose “human tumor VS. xenograft” pairs is maximum. After GSE35144 is removed, the number of “common differentially expressed genes” is 25. But there aree still 16 genes overlapped with previous 48 genes (S1B Fig). Then we did clustering analysis to sample with new selected genes (S2 Fig). The highly similar clustering results suggest that the cluster of “human tumor VS. xenograft” pairs are not caused by some dominant data. Our results demonstrate that multiple cancer types share some similar expression changes in “human tumor VS. xenograft” pairs.

### Functions of differential genes in PDX and potential association with cancer drugs

Since 48 genes are differentially expressed in multiple PDXs, their functions are important to understand the engraftment from cancer patient biopsy to mice. Functional enrichment analysis show that 48 genes are enriched in “extracellular matrix”, “ECM-receptor interaction”, “immune response” or similar terms, and many genes encode signal or secreted proteins (Table 2). We defined a robustness score for the enriched function, the percentage that this function is significantly enriched (Benjamini P-value < 0.01) when analyzing differential gene sets in each “human tumor VS. xenograft” pair. High scores indicate these enriched functions are supported by most of the single datasets. Extracellular matrix is a major component of stromal cells, and plays an important role in tumor microenvironment [22]. Differentially expressed genes in extracellular matrix are consisted with the previous report that human stromal cells were substituted by mice stromal cells [14]. Immune response is a frequent event during allotransplantation and xenotransplantation. Although immune-deficient mice are used to avoid the rejection of human tumor tissue, some human immune-related genes are differentially expressed.

**Table 2:** Functional enrichment of differentially expressed genes in “human tumor VS. xenograft”.

Term	Number of genes	Benjamini P-value	Robustness score^a^
signal	35	1.34E-14	80.5%
secreted	22	3.12E-09	80.5%
extracellular matrix	9	1.50E-06	68.3%
ECM-receptor interaction	5	4.17E-03	56.1%
defense response	11	6.35E-04	75.6%
inflammatory response	5	3.21E-04	73.2%
immune response	16	3.02E-08	75.6%

^a^) The percentage that this function was significantly enriched (Benjamini P-value < 0.01) when analyzing differential gene sets in each “human tumor VS. xenograft” pair.

Among 48 genes, 18 genes have interacting drugs in public databases and 12 genes have been reported to be drug targets (S2 Table). C1QB and C1QC encode a major constituent of the human complement subcomponent, which are targets of 18 drugs. CD14, FCGR3A, FCGR3B, HLA-DPA1, HLA-DPB1, and HLA-DRA play roles in the immune system. CCL2 is one of cytokine genes, involved in immunoregulatory and inflammatory processes. Collagen genes COL1A2 and COL3A1 are the main structural proteins in extracellular matrix and in connective tissue. MMP12 and VCAM1 participate in extracellular matrix organization. Fig 3B shows the number of interacting genes for each drug. For example, alemtuzumab has interaction with four differentially expressed genes. Although these interactions may not really occur, researchers should pay more attention to them when PDX is used to evaluate drug effect, especially when the drug is supposed to have potential interactions with these 18 genes.

### PDX mouse model shows advantages in transcriptome comparison

**Fig 4:**
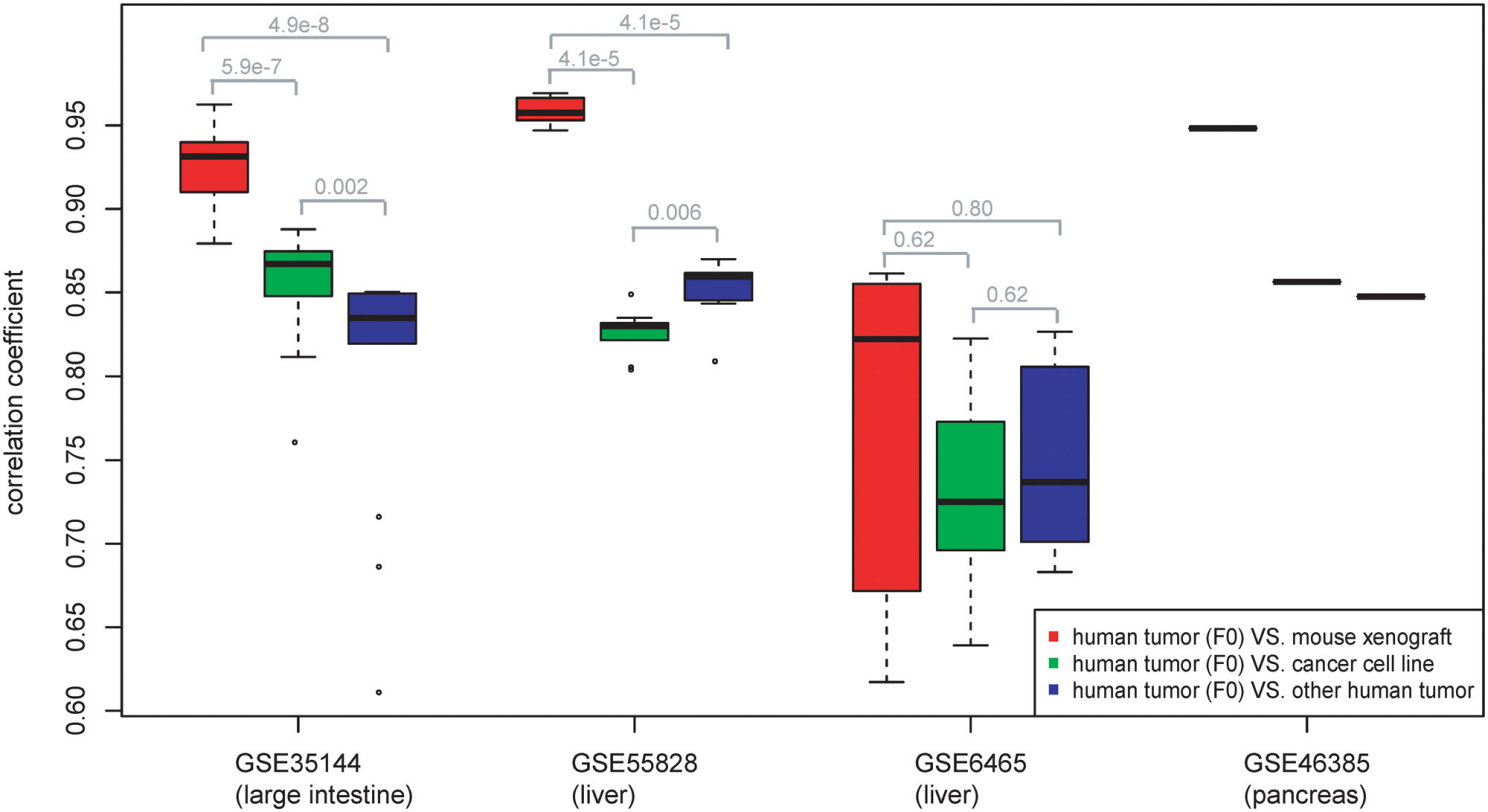
Comparison of human cancer patients (F0) and cancer models from three sources: 1) Mouse xenograft that’s derived from the same patient (“red”); 2) Cancer cell lines in GDSC dataset (“green”); 3) Other human solid tumors in GSE2109 dataset (“blue”). The Y-axis is the maximum correlation coefficient of gene expression profiles between F0 and cancer models.

Before PDX is widely used as preclinical models, tumor-derived cell lines have been used for many years for evaluating candidate anticancer agents [23]. Another way is inferring clinical response from tumor patients with similar pathology and molecular features, such as expression profile and genomic variations [24–26]. Here we compared the gene expression of cancer patients with three kinds of models: PDX, tumor cell lines, and other patients with same cancers. We focused on large intestine, liver, and pancreas cancers that had available expression data (S3 Table). SRCCs between cancer patients (F0) and samples in different models were calculated to get the most similar sample with max SRCC. Boxplots of SRCCs are shown in Fig 4. Cancer patients are more similar with itself derived xenografts in all datasets. Results from another two kinds of models are not consistent. Patients in GSE35144 have higher SRCC when compared to cancer cell lines, while patients in GSE55828 have higher SRCC when compared to other primary cancer patients (Fig 4). This reflects the limitation of the current cell-line panel and primary-tumor dataset, such as loss of microenvironment, incomplete representation of tumor diversity. Therefore, mice xenografts built from original patients best present the characteristics of tumors and PDX mice are better cancer models than cell lines.

### Effect of expression change in drug response

**Fig 5:**
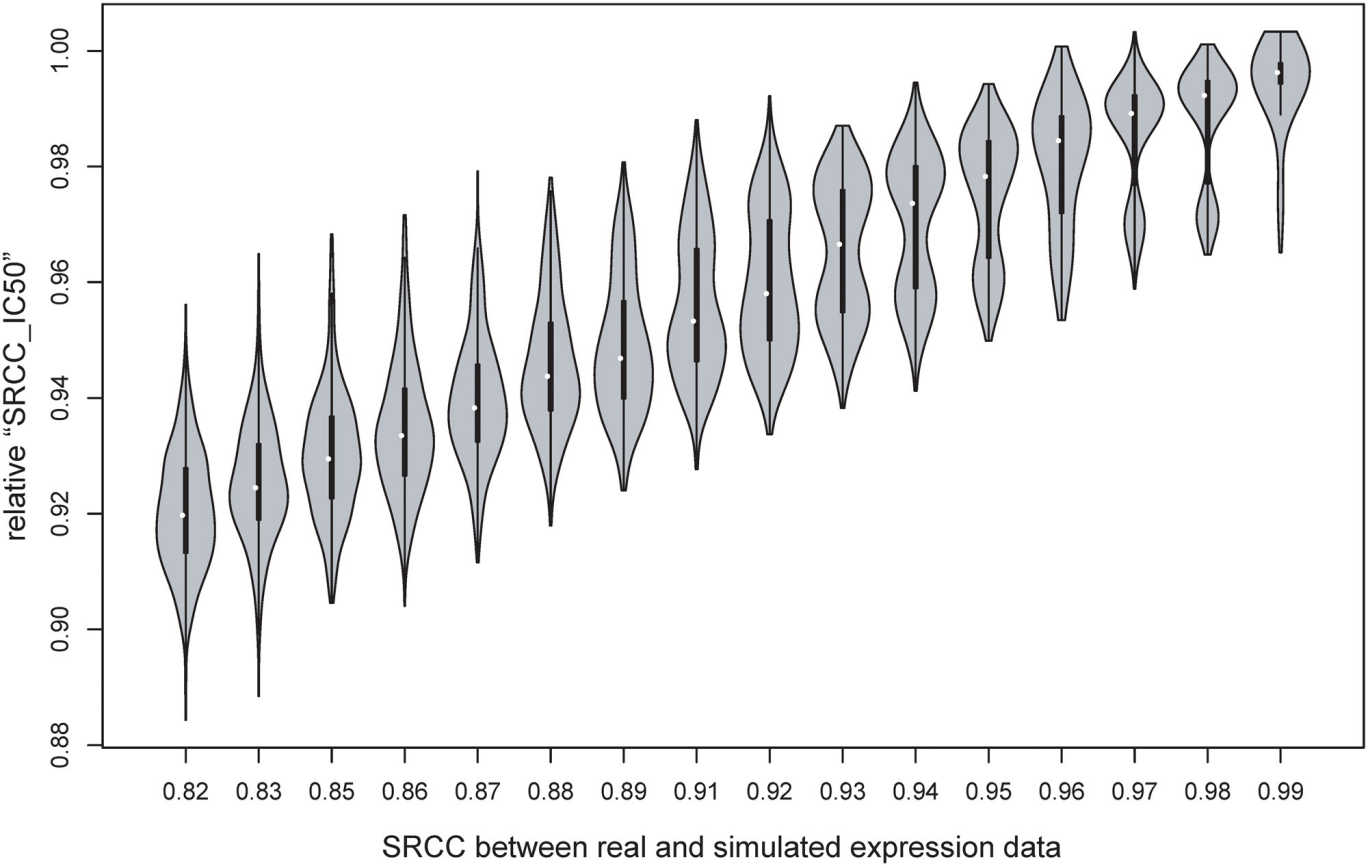
Effect of gene expression change on predicted cisplatin IC50. SRCC between simulated and real expression data was used to measure expression change. IC50 of simulated datasets were predicted by a ridge regression model. The relative “SRCC_IC50” for simulated datasets were used to measure the effect of expression change on drug sensitivity.

One of the most important applications of PDX is personal drug selection for cancer patients. Although PDX is highly similar with patient biopsy, it is still necessary to understand the effect of slight expression change (between patient and PDX) on drug sensitivity. Since there are no drug response data from both patients and PDXs in public domain, we therefore generated multiple simulated datasets from GDSC data and used a computational model to predict the sensitivity of cisplatin (IC50 value). SRCCs between simulated and real expression data were used to measure expression change, simulating the change from tumor biopsy to PDX. The relative “SRCC_IC50” was used to measure the effect of expression change in drug sensitivity (seeing method section for detailed description). The simulation results are illustrated in Fig 5. Generally, larger expression change will result in lower consistency in drug response. When the expression correlation between patient and PDX decreases from 1 to 0.82, the mean correlation of drug sensitivity decreases 8% (from 1 to 0.92). As mentioned above, the median SRCC of “human tumor VS. xenograft” is 0.94. The corresponding similarity in drug responses is 0.97, thus 6% expression change in PDX results in 3% difference in drug sensitivity. Due to the complexity of drug response, the exact numbers in the simulation results may vary slightly in reality, while the trend should be same between simulation and reality. Therefore, drug sensitivity got from PDX to some extent recapitulates the real drug sensitivity in patient tumor.

## Discussion and Conclusion

Cancer patient derived xenografts have been widely studied in clinical cancer biology, due to its valuable application in evaluating anticancer drugs and in personal medicine. Previous studies focus on the methods of successfully building PDXs and improving engraftment rate. Some work compared the morphological and molecular feature of patient tumors and mouse xegnorafts, and proved that PDX preserved patient tumor characteristics. However, such conclusions were obtained from a specific cancer type and a small amount of mouse xenografts. Accumulated PDX expression data allows more comprehensive comparison of xenografted tumors with primary patient tumors.

We performed a meta-analysis of PDX transcriptome, covering 8 cancer types, 58 cancer patients, 48 early (< = 3) PDXs, 36 distant PDXs and 19 PDXs with unknown passages. Our results confirmed that PDX was very similar with patient tumors (median SRCC = 0.94), and their similarity was higher than the similarity with cell lines or other patients in same cancer type. Expression change of PDX mostly occurred in the engraftment progress of human tumors into mice. The combining transcriptomic results identified 48 genes that were changed in more than half cancer patients with different cancer types. These genes participate in tumor microenvironment and immune response. A simulation study showed that 6% expression change in PDX was accompanied by 3% difference in drug sensitivity. Due to the complexity of drug response in reality, the exact numbers in the simulation results may be inaccurate in real drug response. However, the trend should be same between simulation and reality: larger change in expression level will result in lower consistency in drug response.

Animal model is similar to human condition, but they are not exactly same. Although animal model may have some limitations due to the expression or regulation difference between animal and human, animal experiments have been widely used to help understanding human biology. Therefore, we demonstrated that PDXs is so far the best cancer model and quality control is necessary during engraftment and over subsequent passages: monitoring the expression similarity between PDX and patient tumor, checking the change of biologically important genes, et al. Our findings are helpful for the development and application of PDX models.

Currently, gene expression microarray is the main technology which is used in PDX study. In the future, other technologies will be more frequently employed to build the complete molecular profile of PDX, such as RNA sequencing, SNP array, whole-genome/exon sequencing, and methylation sequencing. These information will more accurately illustrate the characteristics of PDX such as mouse contamination in PDX tumor, PDX-specific mutations and subclones due to tumor heterogeneity. More and more PDX models will be accumulated and a large “PDX—drug sensitivity” database will be built. Doctors could select suitable drugs for cancer patients based on the drug response data from self-derived PDX or similar PDXs in the database. Studies of PDX will promote personalized cancer therapy and new drug discovery into a higher level.

## Supporting Information

S1 Fig Common differentially expressed genes in PDX mice. (A) Venn diagram of genes. Each time we removed one dataset, and selected the common differential genes from the remaining four datasets. (B) Overlap of genes when using all datasets or removing GSE35144.
(TIF)

S2 Fig Expression heatmap of common differentially expressed genes in PDX mice. Gene were selected by A) using five datasets, B) removing GSE15240, C) removing GSE46385, D) removing GSE35144, E) removing GSE55828, and F) removing GSE57491.
(TIF)

S1 Table Differentially expressed genes in “human tumor VS. xenograft” and “xenograft VS. xenograft” comparisons. (XLS)

S2 Table A list of 48 genes that were differentially expressed in more than half “human tumor VS. xenograft” pairs. The interacting drugs and drug-targets were obtained from DrugBank and “cancerresource” databases.
(XLSX)

S3 Table Three kinds of cancers that have PDX expression data in GEO database, more than 7 cell lines in GDSC dataset, and more than 7 tumor samples in GSE2109. (XLSX)
