# Supplementary figures and images for: Integrated Analysis of Transcriptome in Cancer Patient-Derived Xenografts
Source: PLoS One. 2015 May 7;10(5):e0124780. doi: 10.1371/journal.pone.0124780 (PMC4423839; doi:10.1371/journal.pone.0124780)

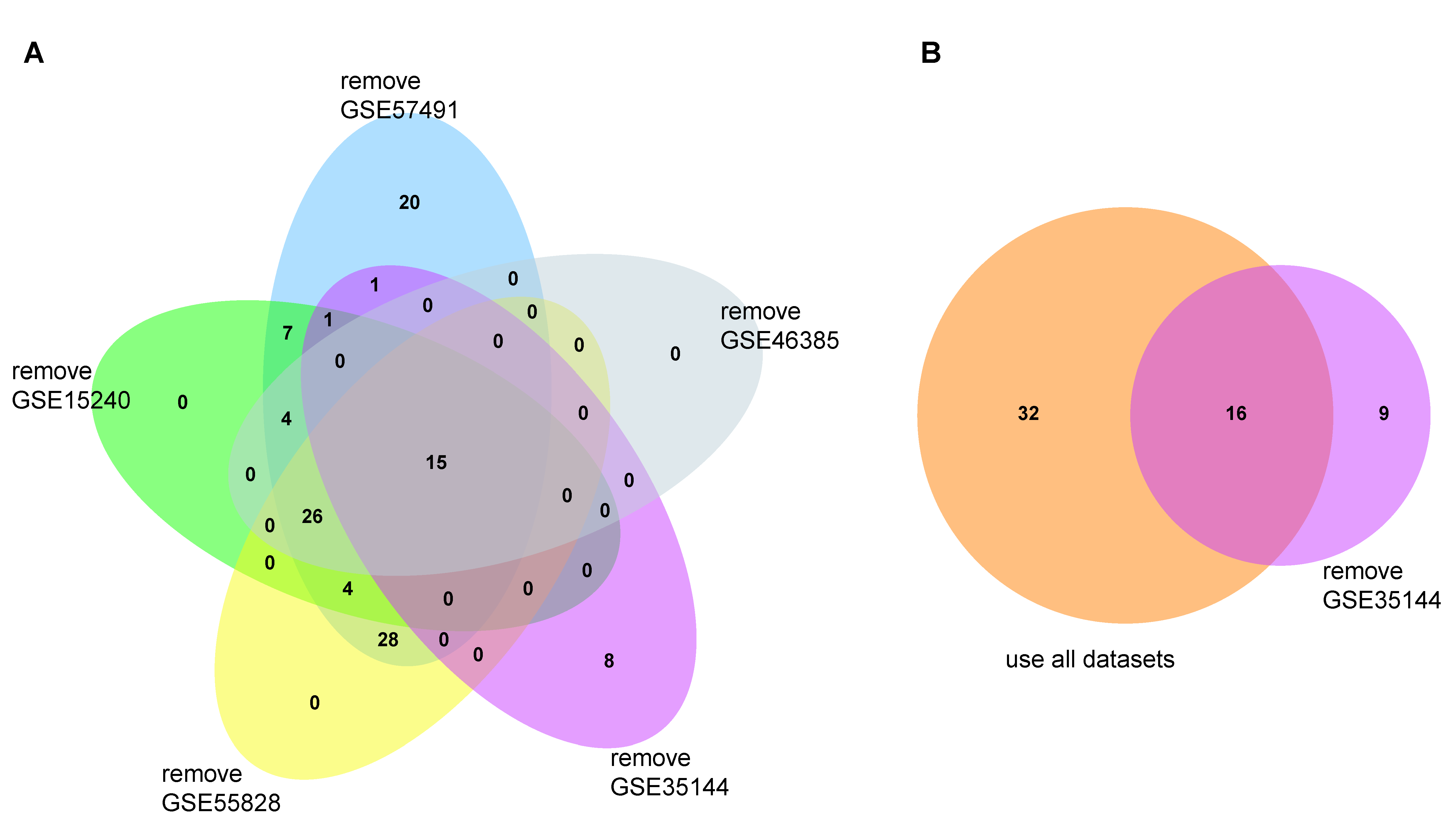

Supplement: S1 Fig — (A) Venn diagram of genes. Each time we removed one dataset, and selected the common differential genes from the remaining four datasets. (B) Overlap of genes when using all datasets or removing GSE35144. (TIF) [file pone.0124780.s001.tif]
